# Do White and Black People Truly View the Police Differently? Findings from a Study of Crime Hot Spots in Baltimore, Maryland

**DOI:** 10.1007/s12103-025-09795-x

**Published:** 2025-02-01

**Authors:** Kiseong Kuen, CJ Appleton, David Weisburd, Clair V. Uding

**Affiliations:** 1 School of Criminology and Criminal Justice, Griffith University, Building G06 Room 3.37, 1 Parklands Drive, Southport, Gold Coast, QLD 4215 Australia; 2 Department of Criminology, Law and Society, George Mason University, Fairfax, VA USA; 3 Institute of Criminology, Hebrew University of Jerusalem, Mt. Scopus, Jerusalem, Israel; 4 Department of Criminal Justice and Sociology, University of Wyoming, Laramie, WY USA

**Keywords:** Race, Perceptions of the police, Procedural justice, Police effectiveness, Police legitimacy, Propensity-score matching

## Abstract

While numerous studies demonstrate that Black individuals have more negative perceptions of the police than their White counterparts, few have simultaneously examined racial differences in perceptions of procedural justice, police effectiveness, and legitimacy. Additionally, limited research has rigorously examined the relationship between race and perceptions of the police while carefully accounting for potentially relevant factors that could influence this relationship. Using unique survey data largely drawn from crime hot spots in Baltimore, Maryland, we examined the differences between White (*n* = 500) and Black (*n* = 2,452) individuals’ perceptions of procedural justice, police effectiveness, and police legitimacy. Furthermore, by conducting propensity-score matching on White and Black residents in our data, we compared perceptions of the police between 394 pairs of similarly situated Black and White residents who were matched based on demographics, victimization, offending, self-control, recent experiences with the police, perceived police presence, and street environments. Results indicated that while Black people have more negative perceptions of procedural justice than White people, they do not hold different perceptions regarding police effectiveness and obligation to obey. These findings hold even when comparing the matched White and Black people. Our findings suggest a nuanced relationship between race and perceptions of the police.

**Supplementary Information:**

The online version contains supplementary material available at 10.1007/s12103-025-09795-x.

## Introduction

American policing has faced a *crisis of legitimacy* in the 21st century (Lum, 2021; Worden & McLean, 2017). Media outlets frequently highlight a stark racial divide in perceptions of the police within the United States (Peck, 2015). Indeed, a large body of research on public perceptions of the police has reported that Black individuals are more likely to hold negative views towards the police than their counterpart White individuals (Brunson, 2007; Drakulich et al., 2023; Hurst & Frank, 2000; Hurst et al., 2000; Johnson et al., 2017; Lum et al., 2024; MacDonald et al., 2007; Peck, 2015; Pickett et al., 2022; Reisig & Park, 2000; Weitzer et al., 2008). Brunson (2007) noted more than a decade ago that “one of the most reliable findings in research on attitudes toward police is that citizen distrust is more widespread among African-Americans than Whites” (p. 73).

However, despite extensive research, few studies have simultaneously examined how racial disparities—or the lack thereof—manifest across various aspects of public perceptions of the police, including procedural justice, police effectiveness, and police legitimacy. Consequently, it remains unclear whether racial disparities are specific to particular dimensions of these perceptions or whether they follow similar patterns across multiple aspects. This is particularly surprising given the growing emphasis on police legitimacy and its antecedents—procedural justice and police effectiveness (Sunshine & Tyler, 2003; Weisburd & Majmundar, 2018; Weisburd et al., 2024a). Addressing this gap is essential, as each dimension provides unique insights into public attitudes toward the police and police-community relations (see Tyler, 2004; Tyler & Jackson, 2014). By not considering these dimensions collectively, previous research may have overlooked critical nuances in the ways White and Black individuals perceive and evaluate the police differently.

Futhermore, it remains unclear whether racial disparities in perceptions of the police primarily reflect differences in individual characteristics (e.g., socioeconomic status, victimization) and place-based factors (e.g., crime, disorder), or alternatively stem from differing group relationships with the police shaped by historical and contemporary patterns of discrimination (MacDonald et al., 2007; Sampson & Bartusch, 1998). For instance, Black individuals are often overrepresented in crime hot spots and tend to have lower socioeconomic status compared to White individuals, yet these fundamental differences have rarely been adequately accounted for in prior research. Research examining racial differences in perceptions of the police has relied on traditional regression models with a limited set of covariates related to perceptions of the police, making them vulnerable to selection bias (for an exception, see MacDonald et al., 2007). Therefore, studies that better address these limitations using a diverse range of relevant covariates and applying more rigorous methodologies are needed to enhance our understanding of the relationship between race and perceptions of the police.

In this study, we first compare the perceptions of White (*n* = 500) and Black (*n* = 2,452) individuals toward the police across various dimensions using the entire sample. We then apply a propensity-score matching (PSM) approach to compare the perceptions of the police among 394 pairs of similarly situated White and Black residents, matched on a range of individual characteristics, recent victimization and offending history, recent experience with the police, perceptions of police presence, and attributes of the streets where they live. Specifically, drawing on extensive survey data primarily collected from crime hot spots in Baltimore City, Maryland, we rigorously examine racial disparities—or the lack thereof—in perceptions of procedural justice, police effectiveness, and police legitimacy (i.e., obligation to obey the police). By focusing on marginalized communities frequently exposed to crime, disorder, and strained relations with law enforcement, this study provides a nuanced understanding of public perceptions of the police in contexts that are significant yet rarely addressed in prior research.

## Importance of Obligation to Obey, Procedural Justice, and Police Effectiveness as Concepts of Citizen Perceptions of the Police

Because of their significant theoretical and policy implications for contemporary policing, recent research into citizen perceptions of the police centers around police legitimacy and its core antecedents—procedural justice and police effectiveness (see Sunshine & Tyler, 2003; Weisburd & Majmundar, 2018). Police legitimacy can be defined in different ways. However, it generally refers to a citizen’s belief that “the police are entitled to call upon the public to follow the law and help combat crime, and that members of the public have an obligation to engage in cooperative behaviors” (Tyler, 2004, pp. 86–87). The police are considered legitimate when the public believes their decisions and actions to be right and that their authority should be obeyed (Tyler, 2004). Public perceptions of legitimacy are essential because they determine the extent to which the public is willing to cooperate, assist, and empower the police (Kuen et al., 2024; Madon et al., 2017; Murphy et al., 2014; Sunshine & Tyler, 2003; Tyler & Jackson, 2014). As such, legitimacy plays a key role in the overall effectiveness of the police in responding to and reducing crime.

Procedural justice and police effectiveness have gained substantial scholarly and policy attention as the literature on police legitimacy has identified them as key precursors to legitimacy. In general, procedural justice refers to how police act during their interactions with people. The concept of procedural justice is built on the premise that citizens’ perceptions of the police stem from how they, or those they see, are treated by the police (Madon et al., 2017; Tyler, 2004). On the other hand, police effectiveness refers to the public’s perceptions that the police successfully reduce crime and address other problems according to the public’s needs (Jonathan-Zamir & Weisburd, 2013; Sunshine & Tyler, 2003). Police effectiveness is a more utilitarian concept than procedural justice because it mainly focuses on outcomes that police can address rather than the processes by which police behave. Of the two concepts, procedural justice is more strongly associated with police legitimacy in most contexts and countries (Jonathan-Zamir & Weisburd, 2013; Kuen et al., 2024; Murphy et al., 2014; Sunshine & Tyler, 2003; Tyler & Jackson, 2014; Wolfe et al., 2016).

While debates continue over the conceptualization and operationalization of procedural justice, police effectiveness, and obligation to obey the police (Kuen, 2024; Tankebe, 2013; Tyler & Jackson, 2013, 2014), these concepts are central to understanding public perceptions of law enforcement (see Weisburd & Majmundar, 2018). In democratic societies, it is imperative that police treat citizens in a procedurally fair manner. Police effectiveness is also crucial, as addressing crime problems and ensuring public safety are primary responsibilities of law enforcement. Citizens’ feelings of obligation to obey the police are vital (regardless of whether it is a component or an outcome of legitimacy; see Tankebe, 2013) because it serves a self-regulatory function (Tyler, 1990). Therefore, fostering positive perceptions of these aspects among community members of all classes and races is a major task for police. However, little research has examined these diverse dimensions in the context of racial differences between White and Black individuals. To address the gap, our study focuses on perceptions of procedural justice, police effectiveness, and obligation to obey the police as outcomes in examining racial differences in citizen perceptions of the police.

## Perspectives on Racial Differences (or the Lack Thereof) in Perceptions of the Police

There are several perspectives about what potentially causes differences (or the lack thereof) in perceptions of the police between Black and White individuals. The first perspective—the group-position model—suggests how Black individuals’ collective experiences, rooted in their marginalized group position in U.S. society, shape their negative attitudes toward the police (see Bobo & Hutchings, 1996). This perspective posits that race is one of the most unique and important factors shaping one’s perception of the police in the United States (Brunson, 2007; MacDonald et al., 2007), focusing on historical and current-day forms of discrimination and state-sanctioned violence toward Black people. Black individuals’ negative perceptions of the police date back to police oppression experienced by Black people in the pre-civil War era (e.g., enforcement of the Fugitive Slave Act) (Walker, 1980). Police mistreatment and suppression of Black individuals persisted in the post-slavery era, including Black Codes and Jim Crow laws (Bass, 2001; Updegrove et al., 2024). Some commentators argue that this trend has continued with forms of *zero-tolerance policing* in Black communities in the present era as one of the widely used crime-fighting strategies available to the police (e.g., see Fabricant, 2011). From this standpoint, the accumulated history of police suppression and unfair treatment towards Black people in the United States gives form to contemporary negative views and distrust of the police among Black individuals (Pryce & Chenane, 2021; Walker et al., 2000).

Indeed, many mainstream views have tied modern-day phenomena to historic forms of racial injustice (Alexander, 2011; Coates, 2015). Despite the belief by many that we live in a post-racial society (Bhopal, 2018), these scholars draw a line from past to present, arguing that the current role of the police in society may appear different but is still motivated by the pursuit of racial domination (Feagin, 2001; Omi & Winant, 2014). For example, studies investigating stop, question, and frisk tactics in several cities in the U. S. suggest that the police practices have systematically targeted Black people much more than White people (Brunson & Weitzer, 2009; Epp et al., 2014). This line of research has suggested that Black individuals’ negative encounters with the police are not relegated to only those in structurally marginalized places. Studies have found that neither demographic factors (i.e., income, education) nor community-level structural factors (i.e., concentrated disadvantage, crime, disorder) can explain away the differing views of the police between White and Black people (MacDonald et al., 2007; also see Lum et al., 2024; Reisig & Park, 2000; Tyler & Jackson, 2014; Weitzer et al., 2008; Wu, 2014). For instance, Pryce and Chenane (2021) interviewed 77 Black residents from three community types—public housing, middle-income, and upper-middle-income communities—in Durham, NC, finding that Black residents in the sample, regardless of the community types, generally believed that they are more mistreated and disrespected by the police than White residents (also see Brunson & Weitzer, 2009).

On the other hand, another perspective emphasizes community structural (or ecological) and personal disadvantage, arguing that perceptions of the police are multifaceted and shaped primarily by factors other than race itself. This framework suggests that racial disparities in perceptions of the police could largely reflect differences in individual characteristics (e.g., socioeconomic status, victimization), direct experiences (e.g., unsatisfactory experience with the police), or place features (e.g., crime, disorder) where individuals live (Sampson & Bartusch, 1998). Supporting this standpoint, several studies have found that public perceptions of the police are not significantly different between races when other important individual-level characteristics or place contexts are considered (e.g., see Metcalfe & Bolaji, 2024; Wheeler et al., 2020; Wolfe et al., 2016; Wu et al., 2009). Indeed, Cao and Wu (2019) suggest, in a recent review of the relationship between race and confidence in the police, that the effect of race is weak and not significant in the literature when other key covariates, such as contact with the police or perceptions of the neighborhood, are considered. This standpoint holds that Black individuals are more likely to have lower socioeconomic status or live in neighborhoods with more social problems than their White counterparts. These conditions, in turn, may lead to negative attitudes towards the police, as they tend to have higher crime rates, increased police activity, and increased coercive police behavior (see Nouri, 2021).

Weitzer (1999) suggests that Black residents in marginalized communities tend to perceive that they are being targeted and mistreated by the police, while they also find that Black residents living in middle-class neighborhoods tend not to perceive such racial bias or mistreatment by the police in their community. In addition, Anderson (1999) argues that Black residents living in neighborhoods with concentrated disadvantages tend to perceive that policing in their community is ineffective. This perception stems from Black residents believing that the police do not respond appropriately to their need for safety from serious crimes or that police focus on issues irrelevant to their safety, such as minor drug issues. For instance, research has shown that while Black people are overpoliced in marginalized communities for minor issues such as drug use (King & Mauer, 2006; Lum, 2021), they are at the same time under-policed for serious crimes such as homicide (Lum, 2021). Recently, Boehme et al. (2022) found that Black people were more likely to perceive over- and under-policing than White people. However, the racial difference became non-significant when community-level factors such as collective efficacy and concentrated disadvantage were accounted for.

In short, while the group-position model highlights the enduring influence of racialized group dynamics and historical oppression, the structural/personal disadvantage framework focuses on individual and place-based factors that can further exacerbate negative perceptions of law enforcement. Importantly, these perspectives are not mutually exclusive; rather, they complement each other by offering distinct yet interconnected insights into the complex mechanisms shaping racialized attitudes toward the police. Drawing on these perspectives, this study examines whether White and Black individuals’ perceptions of the police differ, even when key individual and place-level factors were accounted for, or if these perceptions align more closely with broader systemic patterns that transcend individual and contextual differences captured in the data.

## Current Study

In the current study, we compare perceptions of the police between Black and White residents, taking advantage of a large survey conducted in Baltimore, Maryland, a city where Black individuals make up a larger proportion of the population than White individuals. Moreover, using a PSM technique, the current study aims to compare perceptions of the police between Black and White residents who share similar individual characteristics, recent experiences with the police, police presence, and street environments where they live.

## Data Collection

The data were obtained from a study of crime hot spots in Baltimore City, Maryland, initiated in 2012 funded by the National Institutes of Health (Weisburd et al., 2011). Baltimore City has a substantial population of color, with 64% of residents identifying as Black and a poverty rate of 24%, significantly higher than the national average of 15.1% (US Census Bureau, 2015). At the beginning of the project, Baltimore City’s violent crime rate was nearly four times the national average (City Data, 2012). Police-community relations in Baltimore have been strained for decades, exacerbated by zero-tolerance policing practices that disproportionately affect Black residents, as well as persistent racial segregation and widespread poverty (Hinkle et al., 2023; White et al., 2018). Additionally, Baltimore has a significant history of police violence, illustrated by the numerous lawsuits filed between 2011 and 2014 over brutality and false arrests, which led to settlements that totaled $5.7 million (Puente & Donovan, 2015).

Our analyses are based on residential surveys on 449 street segments (both block faces on a street between two intersections). A multistage clustered sampling based on street segments started with a population of 25,045 street segments in the city. Because of its focus on residential streets, the sampling procedure used in this dataset only included street segments with 20 or more occupied dwelling units. This procedure decreased the population of street segments to a sample of 4,630 residential street segments. Using calls for service data for 2012 from the Police Department of Baltimore City, streets were classified as crime hot spots and non-hot spots: combined drug/violent crime hot spots, violent crime hot spots, drug crime hot spots, cold spots, and cool spots.^1^

Each crime hot spot in the sample (violent, drug, and combined violent/drug) was within about the top 3% of all city segments in terms of either violent or drug crime calls for service as of 2012.^2^ A comparison group of “non-hot spot” street segments was also sampled and divided into cold spots, defined as streets with three or fewer calls for drug or violent crime (i.e., relatively crime-free streets), and cool spots for the remaining non-hot spot street segments with four or more calls, but less than the hot spot thresholds (i.e., neither hot spots nor crime-free streets). The final sample of street segments consisted of 55 combined drug and violent crime hot spots, 126 violent crime hot spots, 121 drug crime hot spots, 100 cool spots, and 47 cold spots. The oversampling of crime hot spots in Baltimore City provides unique insights into the perceptions of police among individuals living in marginalized communities frequently exposed to crime, disorder, and contentious relations with law enforcement—a population rarely examined in prior research on public perceptions of procedural justice and police legitimacy (Kuen, 2024; see also Koper et al., 2022).

After the sample of street segments and the sampling frame of occupied dwelling units were identified, well-trained field researchers conducted door-to-door residential surveys between August 2013 and June 2014. Residential dwelling units were randomly sampled on the sample street segments. Those selected were contacted by field researchers who then interviewed the first adult resident willing to participate (at least 21 years old) who had lived at the residence for at least three months. Face-to-face surveys were conducted with Scantron bubble forms that were read to the participants and completed by the field researcher. The survey took about 20–25 minutes, and respondents were compensated $15 for their participation. The contact and cooperation rates for the survey were 71.2% and 60.5%, respectively, which are above-average results for door-to-door surveying (Babbie, 2020). Finally, a total of 3,738 residential surveys were completed, with an average of eight surveys completed on each street segment. It is important to note that the Hispanic (*n* = 172) and other race groups (*n* = 211) were excluded from the current study since their sample sizes were too small to be appropriately matched with White or Black residents in the sample.^3^ After listwise deletion for missing data (*n* = 403), a total of 2,952 surveys (500 White residents and 2,452 Black residents) were used for our analyses.^4^ After the matching, our final sample included 394 matched pairs of White and Black residents. The analytic strategy section addresses the full process of PSM in detail.

## Measures

### Dependent Variables

In this study, we focused on three dependent variables capturing citizen perceptions of the police—procedural justice, police effectiveness, and obligation to obey the police.^5^ Although there are ongoing debates surrounding what concepts and measures most appropriately capture these three constructs, the items on the survey have been used extensively in the literature (Sunshine & Tyler, 2003; Wolfe et al., 2016). For all three variables, items used a 4-point Likert scale with response options of 1 = *strongly disagree* to 4 = *strongly agree*.

The *procedural justice* scale capturing perceptions of how the police treat citizens was made up of three items (α = 0.85), (1) *“*Police officers treat people fairly”; (2) “In general, the police care about your problems on your block”; and (3) *“*In general, police officers treat people with respect.*”* The *Police effectiveness* scale referring to the performance of the police in addressing crime problems and regulating orders is comprised of three items (α = 0.71): (1) *“*In general, the police do a good job preventing crime”; (2) *“*The police do a good job controlling drug activity*”*; and (3) *“*The police do a good job enforcing traffic laws.” In this study, *obligation to obey the police—*the feeling that the police are entitled to be obeyed*—*is measured with a single item, “If a person is doing something wrong and a police officer tells them to stop, they should stop even if they think what they are doing is legal.” 6

### Treatment Variable

The treatment variable, *race*, is captured by a dummy variable (1 = White, 0 = Black) identifying whether the survey respondent is Black or White.

### Balancing/Control Variables

Stuart (2010) suggests that balancing PSM variables should be associated with outcome variables. A large body of literature suggests that one’s demographics (Hurst et al., 2000; Reisig & Parks, 2000; Sampson & Bartusch, 1998; Wu et al., 2009), victimization (Wu et al., 2009), offending (Lee et al., 2010), low self-control (Wolfe, 2011), recent experience with the police (Skogan, 2006; Weisburd et al., 2024a), perceptions of police presence (Koper et al., 2022; Weisburd et al., 2024a; Yesberg et al., 2023), and perceptions of the place where they live in (Gau et al., 2012; Reisig & Parks, 2000; Weisburd et al., 2024a; Yesberg et al., 2023) affect perceptions of the police. Thus, in the current study, the propensity-scores on race were estimated using such factors (and related factors) to isolate the racial disparities in perceptions of the police between White and Black individuals.

Demographic variables included *gender* (1 = female, 0 = male), *age*, *education* (three dummy variables representing high school diploma, some college, and bachelors or higher with a reference group of less than high school), *homeownership* (1 = homeowner; 0 = renter), *work status* (two dummy variables representing working part-time and not working/‘other’ with a reference group of working full-time), and *marital status* (1 = married, 0 = not married which includes being single, divorced, separated, or widowed). We also included *victimization* in the past year (1 = yes, 0 = no), *offending* in the past year (1 = yes, 0 = no)^7^ and low self-control (see Appendix A for a complete list of items and Cronbach’s alpha for the construct).

Recent experiences with the police were measured by *filing complaint about the police* while living on the street (1 = yes, 0 = no), *being arrested* in the past year (1 = yes, 0 = no); and *having called the police to report a problem* in the past year (1 = yes, 0 = no). Perceived police presence was measured with *perceived presence of walking officers* on the street segment where they live (1 = less than once a month, 2 = a few times a month, 3 = a few times a week, 4 = everyday) and *perceived presence of police cars* on the street segment (the number of police cars driving on the block on an average day).^8^

Lastly, to capture perceptions of the street characteristics possibly related to policing, we measured *perceptions of social disorder*, *physical disorder*,* collective efficacy* (i.e.,* social cohesion and willingness to intervene*), and *fear of crime* (see Appendix A for a complete list of items and Cronbach’s alpha for each construct). It is important to note that *street segment type* categorized by calls for service related to crimes (four dummy variables representing cool spot, drug hot spot, violent hot spot, and combined drug/violent hot spot with a reference group of cold spot) was also included to control for the objective level of crime on the street segment. Finally, we included the number of years living on the street segment.

## Analytic Strategy

While we present raw differences in perceptions of the police between White and Black residents, our primary approach for accounting for key covariates is propensity-score matching (PSM). PSM is a statistical method to reduce treatment selection bias by creating a sample in which observed confounding factors are balanced between the treatment and control groups. Although PSM does not guarantee causality and is subject to omitted variable bias, it does provide several advantages, in terms of internal validity, over the traditional regression approaches (Stuart, 2010; Weisburd et al., 2022b). Perhaps most important is its approach of focusing on unbiased predictions of the propensity rather than of specific variables in a model. This means that even if there is a bias from unobserved variables, if balancing factors included in the PSM model are strongly associated with the unobserved factors, the unobserved variables bias will be reduced (Stuart, 2010; Weisburd et al., 2022b).

In turn, the PSM technique is more interpretable and less likely to be susceptible to errors in model assumptions than traditional regression analyses (Ridgeway, 2006). PSM can control for numerous confounding factors simultaneously without specifying a particular functional form for the relationship between the variables. This feature makes PSM particularly useful when there are many confounding factors or when the relationships between the factors are complex. For example, Rubin (2001) suggests that when the actual relationship between covariates and the outcome variable is even moderately non-linear in the data, linear regression adjustment (without appropriate matching) can increase biases in the estimation of treatment effect (also see Stuart, 2010). The biases are especially serious when the means and variances of the covariates are very different between the treatment and control groups (Rubin, 2001). It is important to note that propensity score techniques have been widely used to examine racial disparities in the field of policing (MacDonald et al., 2007; Ridgeway, 2006) and criminal justice more generally (e.g., see Higgins et al., 2013).

In this study, the PSM technique enabled us to compare Black and White residents’ perceptions of the police with a procedure that guarantees the same distribution of features on the observable factors for each group. PSM was implemented using *the psmatch2* package in Stata 18. A logistic regression analysis was used to estimate propensity-scores and remove the effects of confounding factors. More specifically, we used 1-to-1 nearest neighbor matching without replacement, which matches each treatment case to a comparison case with the closest propensity-score (see Weisburd et al., 2022b).^9^ A caliper of 0.2 was used to match Black and White subjects, which helps exclude the subjects for whom the two groups had no similar characteristics on the observed factors used for the matching.^10^ To assess the quality of the PSM procedure, we used a t-test and the standardized bias of the covariates (Rosenbaum & Rubin, 1985). Rosenbaum and Rubin (1985) insisted that standardized bias below 10 indicates a good balance in a covariate.

Additionally, given the nested data structure, where individuals are nested within street segments, we performed multilevel linear regression models to estimate adjusted mean differences in perceptions of procedural justice and police effectiveness. For the unmatched White and Black residents, we accounted only for clustering effects at the street level without including covariates. However, for the matched sample, we included all covariates as well as clustering effects at the street-level.^11^ For obligation to obey the police, an ordinal response variable, multilevel ordered logit models were performed to estimate adjusted odds ratio (OR).^12^ Again, for the unmatched sample, we accounted only for street-level clustering effects, whereas for the matched sample, we included both covariates and street-level clustering effects. In the tables for the main analyses (Tables 2 and 3), we report only the racial differences from the multilevel regressions rather than the full models (for full results of the matched sample, see Appendix B and Appendix C). We also estimated Cohen’s *d* and Cramér’s *V* to show the size of the differences in Black and White residents on the raw scores of the perceptions of the police before and after the matching.

We subsequently conducted two sensitivity analyses. First, following a large body of evidence that procedural justice is the main antecedent of obligation to obey the police (Sunshine & Tyler, 2003; Tyler & Jackson, 2014; Wolfe et al., 2016), we also examined the indirect association between race and obligation to obey the police via procedural justice. Using the Karlson–Holm–Breen (KHB) method, which allows decomposing of the total effect into the direct and indirect (i.e., mediation) effects in non-linear regression models (e.g., ordered logit regression model) (Karlson et al., 2012), we examined the indirect effect of race on obligation to obey the police via perception of procedural justice with the ordered logit regression model for the matched sample. Second, to assess the extent to which unobserved factors affected the robustness of study findings, the current study employed the Rosenbaum bounds approach, a widely used sensitivity analysis for detecting potential bias from unobserved variables (Rosenbaum, 2002). This method enables us to examine to what extent the significant results based on PSM are insensitive to bias derived from the presence of omitted variables.

## Results

### Sample Characteristics Before and after Matching

In Table 1, we provide descriptive statistics on balancing variables for the unmatched full sample as well as the matched sample after PSM. As is apparent, the two groups were substantially different on a number of the factors examined before matching, but the PSM procedure led to balance on the included characteristics. Notably, after matching, the standardized biases of the sample characteristics were all below the standard of 10 that Rosenbaum and Rubin (1985) suggested for evident balance. A series of t-tests with the matched groups indicate no significant differences between White and Black residents on all observed covariates.

**Table 1:** Pre- and post-match differences on balancing variables between white and black residents

	Unmatched				Matched			
% or mean	Whites	Blacks	SB(%)	t-test	Whites	Blacks	SB(%)	t-test
Female	0.54	0.59	−8.5	−1.72	0.55	0.53	4.6	0.64
Age	45.47	44.68	5.0	1.02	46.47	46.47	0.0	−0.00
Education (ref = Less than high school)								
High school diploma	0.19	0.39	−44.0	−8.26***	0.24	0.23	2.3	0.34
Some college	0.19	0.31	−28.5	−5.42***	0.23	0.19	7.7	1.14
Bachelors or higher	0.44	0.10	82.6	19.90***	0.32	0.33	−2.5	−0.30
Homeowner	0.56	0.35	42.2	8.57***	0.51	0.51	−1.0	−0.14
Work status (ref = Full-time)								
Part-time	0.11	0.13	−5.1	−1.00	0.10	0.09	3.1	0.48
Others (e.g., not working, retired)	0.44	0.53	−19.4	−3.89***	0.51	0.52	−2.0	−0.28
Married	0.36	0.20	37.8	8.11***	0.31	0.30	1.7	0.23
Victimization	0.41	0.25	34.4	7.19***	0.37	0.38	−2.7	−0.37
Offending	0.14	0.11	9.5	1.98*	0.13	0.14	−3.8	−0.52
Low self-control	2.13	2.07	14.4	2.88**	2.12	2.12	0.5	0.07
Complaint about the police	0.05	0.07	−10.2	−1.93	0.06	0.07	−3.2	−0.44
Arrested	0.07	0.09	−5.9	−1.15	0.09	0.08	1.9	0.25
Called the police to report a problem	0.49	0.30	38.2	7.88***	0.45	0.48	−5.8	−0.78
Perceived presence of walking officers	1.28	1.60	−37.0	−6.75***	1.32	1.34	−2.0	−0.31
Logged presence of police cars	1.01	1.40	−43.0	−8.65***	1.10	1.15	−5.0	−0.70
Perceptions of social disorder	1.73	1.81	−8.8	−1.69	1.79	1.81	−2.7	−0.36
Perceptions of physical disorder	1.40	1.47	−15.9	−3.10**	1.43	1.44	−4.1	−0.56
Social cohesion	3.67	3.52	23.1	4.65***	3.59	3.57	3.3	0.47
Willingness to intervene	3.73	3.75	−2.9	−0.56	3.68	3.62	6.0	0.80
Fear of crime	2.24	2.24	1.6	0.33	2.27	2.27	1.1	0.16
Years on street segment	11.33	9.85	11.1	2.30*	12.16	12.03	1.0	0.13
Street segment type (ref = Cold spots)								
Cool spots	0.23	0.21	4.9	0.98	0.26	0.26	0.0	0.00
Drug crime hot spots	0.14	0.31	−41.3	−7.61***	0.17	0.17	−0.6	−0.09
Violent crime hot spots	0.27	0.29	−4.2	−0.84	0.31	0.32	−1.7	−0.23
Drug/violent crime hot spots	0.06	0.14	−26.5	−4.79***	0.08	0.06	5.1	0.85

SB = standardized bias; * *p* ≤ 0.05, ** *p* ≤ 0.01, *** *p* ≤ 0.001 (two-tailed).

### Main Results

Table 2 provides the results of the t-test and multilevel linear regression models comparing perceptions of procedural justice and police effectiveness between White and Black residents in our sample, both before and after PSM. First, White residents were more likely than Black residents to perceive that the police treat citizens in a procedurally fair manner, with an effect size of *d* = 0.31 before PSM (Adjusted mean difference [*b*] = 0.16, *p* < 0.001) and *d* = 0.24 after PSM (Adjusted mean difference [*b*] = 0.14, *p* < 0.001). Regarding perceptions of police effectiveness, there was no significant difference between White and Black residents, both before PSM (*d* = 0.02, Adjusted mean difference [*b*] = 0.01, *p* > 0.05) and after PSM (*d* = 0.10, Adjusted mean difference [*b*] = 0.05, *p* > 0.05).

**Table 2:** Comparison of perceptions of procedural justice and police effectiveness between White and Black residents before and after matching

	Before matching				After matching			
	Whites (*n* = 500)	Blacks (*n* = 2,452)	Cohen’s *d*	Adjusted mean difference (SE)	Whites (*n* = 394)	Blacks (*n* = 394)	Cohen’s *d*	Adjusted mean difference (SE)
	Mean (SD)	Mean (SD)			Mean (SD)	Mean (SD)		
Procedural justice	2.74 (0.54)	2.55 (0.63)	0.31	0.16 (0.03)***	2.73 (0.56)	2.58 (0.63)	0.24	0.14 (0.04)***
Police effectiveness	2.65 (0.55)	2.64 (0.58)	0.02	0.01 (0.03)^NS^	2.67 (0.55)	2.61 (0.60)	0.10	0.05 (0.04)^NS^

The Cohen’s *d* values are calculated using the means and standard deviations (SD) from the raw scores; The adjusted mean differences before matching are obtained from multilevel OLS regression models that account solely for clustering effects at the street level, while those after matching are derived from models that account for both clustering effects and all covariates; SE = standard error; *** *p* ≤ 0.001 (two-tailed); NS = not significant at the 0.05 level

According to the multilevel ordered logit regression predicting obligation to obey the police (see Table 3), there was also no significant difference in obligation to obey the police between White and Black residents, both before PSM (Adjusted OR = 1.04, *p* > 0.05) and after PSM (Adjusted OR = 1.05, *p* > 0.05). We could not find a significant direct association between race and obligation to obey the police. Following a large body of literature showing that perception of procedural justice and police effectiveness are the two main predictors of feeling an obligation to obey the police (Sunshine & Tyler, 2003; Tyler & Jacskon, 2014; Wolfe et al., 2016), someone might argue that a model predicting obligation to obey the police is misspecified if the two factors are not accounted for. Thus, we also examined the difference in feeling obligated to obey the police between White and Black residents, accounting for perceptions of procedural justice and police effectiveness in a multilevel ordered logit regression (see Model C2 in Appendix C). The results suggest that there was still no significant difference in feeling obligated to obey the police between similarly situated White and Black residents even after the two factors were accounted for (Adjusted OR = 0.97, *p* > 0.05).

**Table 3:** Comparison of obligation to obey the police between White and Black residents before and after matching

	Before matching				After matching			
	Whites (*n* = 500)	Blacks (*n* = 2,452)	Cramér’s *V*	Adjusted OR	Whites (*n* = 394)	Blacks (*n* = 394)	Cramér’s *V*	Adjusted OR
	n (%)	n (%)			n (%)	n (%)		
Strongly agree	81 (16.2%)	360 (14.7%)	0.04	1.04^NS^	65 (16.5%)	69 (17.5%)	0.07	1.05^NS^
Agree	371 (74.2%)	1,877 (76.6%)			300 (76.1%)	290 (73.6%)		
Disagree	47 (9.4%)	196 (8.0%)			29 (7.4%)	31 (7.9%)		
Strongly disagree	1 (0.2%)	19 (0.8%)			0 (0.0%)	4 (1.0%)		
Total	500 (100.0%)	2,452 (100.0%)			394 (100.0%)	394 (100.0%)		

The Cramér’s *V* values are calculated based on chi-square tests; The adjusted odds ratio (OR) before matching was derived from a multilevel ordered logit regression model that accounts solely for clustering effects at the street level, while the adjusted OR after matching was obtained from a model that accounts for both clustering effects and all covariates; NS = not significant at the 0.05 level

### Sensitivity Analyses

As a sensitivity analysis with the matched sample, we used KHB mediation analysis with bootstrapping (5,000 replications) to investigate the indirect effect of race on feeling of obligation to obey the police via perception of police procedural justice (see Appendix D). The KHB mediation analysis result indicated no significant indirect association between being White and feeling obligated to obey via perceived procedural justice (*b* = 0.06, OR = 1.06, *p* > 0.05). In other words, the significant difference in perceived police procedural justice between White and Black residents did not translate into a significant racial difference in their obligation to obey the police.

Given that the PSM approach cannot fully account for unobserved factors, we also assessed the sensitivity of the significant findings to potential omitted variable bias (see Appendix E). Using a 0.05 significance threshold, the result on perceived procedural justice remained significant with gamma (*Γ*) values of up to 1.27 but became not significant at *Γ* = 1.28.^13^ This indicates that the result on procedural justice would remain robust unless unobserved factors were strong enough to bias the odds of selection into the treatment group by over 27% (Rosenbaum, 2002). Put simply, the significant difference in White and Black residents’ perceptions of procedural justice is robust to unobserved bias unless such bias increases the odds of being in the White group by over 27%.

## Discussion

First, our results demonstrate that White and Black residents had substantively different perceptions of police procedural justice, with Black individuals holding more negative attitudes (MacDonald et al., 2007). Notably, Black individuals viewed the police more negatively than White individuals, even when matched on demographics, victimization, offending, self-control, recent experiences with the police, perceived police presence, and street environments. This may simply reflect the reality that the police treat Black people in a more procedurally unfair way than White people. Alternatively, the racial differences may be driven by the unique worldview of Black individuals, as explained by group-position model (see Bobo & Hutchings, 1996). Unnever and Gabbidon (2011) argue that this worldview, shaped by historical and present-day experiences, create a unique racial lens that informs beliefs and behaviors, especially regarding how racism impacts their lives. From this perspective, past traumatic experiences can influence present-day perceptions of America’s institutions, such as the police, through the collective memory shared by Black individuals (Jean & Feagin, 1998; Pryce et al., 2021). While these factors were not directly measured in this study, we believe that this collective memory may make Black people more sensitive to perceived injustice at the hands of state actors compared to their White people counterparts.^14^

Second, for both the full and matched samples, the findings on police effectiveness suggest that White and Black residents do not differ in their perceptions. These results show that Black people’s relationship with and perspective toward the police may be more nuanced than some would believe (Pryce et al., 2021). Mainstream narratives resulting from recent police protests suggest that Black people have more general dissatisfaction with the police (Edwards & Harris, 2015). However, our findings show that two things can be true at the same time: Black people can perceive the police as similarly effective as their counterpart White people, while simultaneously viewing the police as more procedurally unjust. This finding is plausible because police effectiveness is more utilitarian and instrumental than procedural justice based on normative concerns (see Sunshine & Tyler, 2003; Tyler, 1990; Wolfe et al., 2016). For people’s instrumental concerns about police effectiveness, perceptions of police presence or environmental traits on the street segments where they live (i.e., social disorder) seem to be major driving factors rather than race (see Model B2 in Appendix B).

Lastly, we also found that White and Black residents do not have different feelings of obligation to obey the police, whether they are similarly situated or not. This finding conflicts with our finding on procedural justice. Since a large body of studies has shown that procedural justice is the primary antecedent of obligation to obey the police (Kuen et al., 2024; Madon et al., 2017; Sunshine & Tyler, 2003; Tyler & Jackson, 2014; Wolfe et al., 2016), one might expect that if race is the major driving force of the variation of perception of police procedural justice across individuals, one’s feeling of obligation to obey the police should be different by the race. However, the result from the KHB mediation analysis suggests that a significant difference in White and Black residents’ perceptions of police procedural justice did not lead to different feelings of obligation to obey the police between them in our sample. Black people may be as willing to obey the police as White people because they believe it is the right thing to do, despite their view that the police do not treat them in a procedurally just manner.

Another possible explanation might be that obligation to obey the police does not necessarily capture citizen perception of police legitimacy in Baltimore City. As noted earlier, there is a debate on whether obligation to obey the police captures citizens’ “free consent” to the police—based on their normative decision—as a key indicator of police legitimacy, or if it merely represents “dull compulsion” (see Tyler & Jackson, 2013). Tankebe (2013) argues that the feeling of obligation to obey the police may derive from various sources, one of which is perceived police legitimacy, but people may also feel obligated to obey the police for other reasons. For example, in response to the widespread tensions between the police and citizens, most residents in Baltimore may feel obligated to obey the police to avoid conflicts with the police, regardless of their perceptions of police legitimacy.

The findings of the current study provide two important policy implications. First, this study suggests that it is essential for policing scholars and police practitioners to look beyond individuals’ circumstances and environmental factors when trying to compare the evaluation of police procedural justice between Black and White residents. Black people have a unique history that may be transmuted into their present consciousness through collective memory (Pryce et al., 2021). In turn, race has closely been tied to perceptions of the injustice of the police in American society (Russell, 1998), which manifests tense police–Black community relations in the United States (see MacDonald et al., 2007). Undoubtedly, procedural justice training might help reduce Black people’s mistrust of police procedural unfairness. A recent experimental study conducted by Weisburd et al. (2022a) found strong support for procedural justice training in significantly changing officers’ behavior based on procedural justice principles. Training may want to consider Black citizens’ unique positions and experiences as our understanding of minorities’ views of the police grow and training for police evolves. However, a short period of procedurally fair treatment towards Black people might not be enough to change their beliefs in historically embedded racism and injustice by the police. Considering Black people’s established beliefs in police procedural injustice, more systematic and holistic changes in police practices are needed above the organizational level.

Second, however, it appears that the relationship between race and perceptions of the police has nuance, contrary to what is suggested in most previous studies (e.g., MacDonald et al., 2007). Despite the well-documented conflicts and incidents between the police and Black individuals, our results suggest that Black people do not have more negative perceptions of police effectiveness and feelings of obligation to obey the police than their counterpart White people in Baltimore City. As such, reports from Black residents’ dissatisfaction with police procedural justice should not be summed up as another aspect of a general anti-police sentiment. Such assumptions can foster preconceived notions, such as the belief that Black individuals are inherently distrustful of law enforcement, which can lead to biased policing practices against them. Policymakers and police practitioners should move beyond these oversimplifications and recognize the complex and nuanced relationship between race and perceptions of the police.

### Limitations and Future Directions

Although the current study advances the literature on racial disparities in perceptions of the police, it is important to note several limitations and highlight directions for future research. First, while we examined perceptions among White and Black individuals, we were not able to include comparisons involving Hispanic individuals or other racial and ethnic groups. This limitation stems from the small sample sizes of Hispanics and racial minority groups in our data, which made it difficult to appropriately match these groups with Black and White residents for meaningful analysis. We encourage future studies to collect larger samples of Hispanic populations and other minority groups to better understand how perceptions of the police may vary across a broader range of racial and ethnic groups.

Second, while our analyses using PSM techniques provide several advantages in improving internal validity over traditional regression approaches, our study, like others employing PSM, remains subject to omitted variable bias and cannot guarantee unbiased outcome estimates (Loughran et al., 2015; Weisburd et al., 2022b). We employed the Rosenbaum bounds approach for the result on perceptions of procedural justice to assess how sensitive the significant result is to potential bias from unobserved factors. This method, however, does not quantify the extent of actual bias caused by unobserved factors in PSM. Additionally, although we included a wide range of covariates in our matching procedures, certain relevant factors could have been omitted. For example, while we propose that racial differences in perceived police procedural justice may arise from Black individuals’ unique world view, shaped by their historical and present-day experiences, we were not able to explore this possibility fully due to the absence of direct measures in our data. Other important variables recently highlighted in the literature, such as racial resentment and political ideology, also warrant consideration (Drakulich et al., 2023). Future research should incorporate these variables that could explain racial disparities in views of the police.

Third, our findings might not generalize to other US cities, as attitudes toward the police tend to be more negative in Baltimore City compared to many other cities (White et al., 2018). Additionally, while our focus on crime hot spots provides insights into perceptions of the police among community members who are more likely to have direct and indirect experiences with the police, this focus may also limit the external validity of our findings. Future research should examine the relationship between race and perceptions of the police across cities with differing demographics and crime levels and further investigate whether the racial disparities—or lack thereof—vary by cities and places.

Finally, our measures do not capture the full scope of police legitimacy, procedural justice, and police effectiveness. Notably, the obligation to obey the police is measured using only one item, which limits its ability to fully reflect the concept’s meaning. The other two main components of police legitimacy—trust in the police and moral alignment with the police (see Tyler & Jackson, 2014; Wolfe et al., 2016)—were not included in this study due to data limitations; thus, we were not able to assess potential racial disparities in these components of police legitimacy. Additionally, this study measured procedural justice and police effectiveness using only three items each, which also do not capture the full scope of these constructs. Despite these limitations, our findings offer nuanced insights into the relationship between race and perceptions of the police that have rarely been discussed in prior studies. Future research should replicate this study using more comprehensive measures of these concepts to further explore the nuanced relationship.

## Conclusions

Using extensive survey data primarily gathered from crime hot spots in Baltimore City, Maryland, this study examined racial disparities—or the lack thereof—in perceptions of procedural justice, police effectiveness, and obligation to obey the police among residents of marginalized local communities that regularly encounter crime, disorder, and strained relations with the police. Our findings demonstrate that Black residents have more negative perceptions of procedural justice than White residents, while not holding different views on police effectiveness and obligation to obey the police. This finding implies that either the police still treat Black individuals in a more procedurally unfair way or accumulated historical mistreatment towards Black individuals may drive the difference in perception of procedural justice. Nonetheless, the null findings on the differences in perceptions of police effectiveness and obligation to obey between White and Black individuals suggest that the relationship between race and perceptions of the police is more nuanced than previously believed. Future studies should continue to investigate the nuanced relationship between race and perceptions of the police and provide insights for improving police-community relations, particularly the relationship between Black communities and the police.

## Supplementary Information

Below is the link to the electronic supplementary material.

ESM 1 (DOCX 52.7 KB)

## Data Availability

Data and analysis code are available upon request.

## Footnotes

1 Using a geolocator and shapefile provided by the Baltimore City Police Department, we categorized call types as violent, drug, or other crimes. The match rate was 98.8%.

2 The initial thresholds for drug and violent crimes were set at 18 drug-related CFS and 19 violent CFS, respectively, which represent the top 2.5% of city segments in each category. These values were selected as a midpoint between the commonly used 1% and 5% thresholds for identifying crime hot spots (Weisburd et al., 2024b). To better meet sampling goals for streets with high levels of drug-related or violent crime, the thresholds were adjusted to 17 violent CFS and 16 drug CFS, reflecting approximately the top 3% of street segments in each category. Additionally, to ensure that crime levels were consistently high across the year, CFS had to be distributed over at least six months. For a comprehensive description of the study’s methodology, see https://cebcp.org/wp-content/uploads/2020/07/NIDA-Methodology.pdf.

3 We considered constructing an ‘other race/ethnicity’ group by combining the Hispanic sample with other race groups. However, the ‘other’ group exhibited significant heterogeneity in race and ethnicity (n = 29 Asian residents, 43 Native American or Alaskan Native residents, 130 respondents identifying as ‘something else’ (not further specified), and 3 identifying as multiple races). Therefore, we decided not to combine this group with the Hispanic group to create an ‘other race/ethnicity’ category. Specifically, the 130 responses of ‘something else’ without further specification of race or ethnicity raised concerns about their inclusion in the analyses.

4 The total amount of missing cases for the current study was approximately 10% of the total sample of White and Black residents. Given the relatively small percentage of missing cases and enough statistical power to detect meaningful effects, we decided to use listwise deletion to address the missing data (see Schafer, 1999).

5 Confirmatory factor analysis (CFA) demonstrated that the 2-factor model with the six items of procedural justice and police effectiveness showed an acceptable model fit to the data (χ2 = 66.34, *df* = 8, *p* < 0.001, Root mean square error of approximation (RMSEA) = 0.096, Comparative fit index (CFI) = 0.972, Tucker-Lewis index (TLI) = 0.947).

6 In the original data, we also have two relevant items on the obligation to obey: (1) “I always try to follow the law even if I think it is wrong” and (2) “People should obey the law even if it goes against what they think is right.” However, we decided to leave these items out of the current study because they are mainly about obligation to obey the law rather than obligation to obey the police. We confirmed that using the *obligation to obey the police and law* scale, including the two items about law and one item about police, produced very similar results to the current study (analyses available upon request).

7 Offenses included driving a vehicle under the influence of alcohol, damaged someone else’s property on purpose, taken something that didn’t belong to you, used someone else’s credit card or a personal check to steal something, owned or carried a gun without a license, broken into a home or business to steal something, sold illegal drugs, stolen a car or some other type of motor vehicle, used violence against someone-like in a fist fight or assault, and taken something from someone using violence or the threat of violence.

8 Perceived presence of police cars variable was logged due to its positive skewness in the original form.

9 The nearest neighbor matching was done without replacement since the replacement approach tends to result in lower statistical power from the smaller sample size of the comparison group (Weisburd et al., 2022b). We also conducted 2-to-1 and 3-to-1 nearest neighbor matching, but we found that 1-to-1 nearest neighbor matching produced slightly better balances across covariates. The results from 2-to-1 and 3-to-1 nearest neighbor matching yielded similar results (available upon request). Among several matching techniques (e.g., kernel density matching, stratification matching), nearest neighbor matching was used because Rosenbaum bounds can be estimated in the analysis, while other matching techniques do not allow an estimation of this type of sensitivity analysis. We confirmed that the results from Kernel Density and stratification matching arrived at similar results (available upon request).

10 Following Austin (2011), that examined an optimal caliper width for PSM, we used a caliper of 0.2. However, we also confirmed that using other caliper widths (i.e., 0.1, 0.05, or 0.01) produced similar results on matching and outcome variables (available upon request).

11 Likelihood ratio tests comparing the multilevel linear regression model and one-level linear regression model were significant for both procedural justice ($\:{x}^{2}=7.17,\:p<0.01$) and police effectiveness ($\:{x}^{2}=3.86,\:p<0.05$), meaning that procedural justice and police effectiveness significantly vary across street segment, which demonstrates that multilevel modeling is needed to account for clustering effects of the factors at the street-level.

12 The likelihood ratio test comparing the multilevel ordinal logit model and the one-level ordinal logit model for obligation to obey was not significant ($\:{x}^{2}=0.76,\:p=0.192$). However, following Cheah’s (2009) observation that multilevel modeling produces more conservative and reliable estimates than fixing standard error (i.e., clustered standard error) even when clustering effects are not significant, we conducted multilevel ordinal logit models rather than one-level ordered logit model with clustered standard error. We confirmed that the two approaches produced very similar results (available upon request).

13 Rosenbaum bounds were estimated based on the average treatment effect on treated (ATT).

14 A recent study by Drakulich et al. (2023) emphasizes the importance of exploring why White Americans hold more favorable views of the police rather than focusing solely on why Black Americans have less favorable perceptions. This study found that White Americans’ racial resentment toward Black Americans significantly contribute to racial disparities in perceptions of the police. Although this focus extends beyond the scope of the current study, it warrants further exploration in future research (see the limitations section).
