# Supplementary material for: Do White and Black People Truly View the Police Differently? Findings from a Study of Crime Hot Spots in Baltimore, Maryland
Source: Am J Crim Justice. 2025 Feb 1;50(3):541–64. doi: 10.1007/s12103-025-09795-x (PMC12089163; doi:10.1007/s12103-025-09795-x)
Supplement: Supplementary file 1 — (DOCX 52.7 KB) [file 12103_2025_9795_MOESM1_ESM.docx]

**Appendix A**. Measures of low self-control, fear of crime, social disorder, physical disorder, and collective efficacy

| *Low self-control* (α = 0.70) |
| --- |
| Please tell me whether you strongly disagree, disagree, agree, or strongly agree with the following statements about your personality. |
| a. I do certain things that are bad for me, just because they are fun. |
| b. Pleasure and fun sometimes keep me from getting work done. |
| c. I am good at resisting temptation (reverse coded). |
| d. I often act without thinking through the alternatives. |
| e. Sometimes I can’t stop myself from doing something, even if I know it is wrong. |
| *Fear of crime* (α = 0.78) |
| Please tell me if you strongly disagree, disagree, agree, or strongly agree with each of the following statements about your block. |
| a. You are afraid of being attacked on your block. |
| b. You are worried that someone will break into your home. |
| c. You are worried about drugs on your blocks. |
| d. It is safe for children to play outside on your block (reverse coded). |
| e. In general, it is safe to walk on your block at night (reverse coded). |
| f. Most people think your block is becoming more dangerous. |
| *Perceived social disorder* (α = 0.88) |
| Over the past year, how often do you think the following types of activities have taken place on your block? Do you think these activities take place every day, a few times a week, a few times a month, or less than once a month? |
| a. People arguing or fighting on your block. |
| b. Groups of kids hanging out, causing problems. |
| c. People drinking alcohol in public. |
| d. People acting drunk or high on your block. |
| e. People making too much noise late at night. |
| f. People selling drugs outside. |
| *Perceived physical disorder* (α = 0.75) |
| Now I’d like to ask you some questions about the physical appearance of your block. For each question, tell me if there are none, one or two, or many? |
| a. Buildings with broken windows on your block. |
| b. Places on your block where graffiti is a problem. |
| c. Vacant lots on your block. |
| d. Abandoned or boarded up buildings on your block. |
| e. Places on your block where litter and broken glass are a problem. |
| *Collective efficacy* |
| *Social cohesion and trust* (α = 0.76) |
| For each of the following statements, please tell me if you strongly disagree, disagree, agree, or strongly agree. |
| a. People on your block are willing to help their neighbors. |
| b. Neighbors do NOT talk to each other on your block (reverse coded). |
| c. In general, people on your block can be trusted. |
| d. People on your block usually do NOT get along with each other (reverse coded). |
| e. People on your block do NOT share the same values (reverse coded). |
| f. Neighbors watch out for each other on your block. |
| *Willingness to intervene* (α = 0.86) |
| Please tell me if it is very unlikely, unlikely, likely, or very likely that the following things would happen on your block. |
| a. If some kids were skipping school and hanging out on your block, how likely is it that your neighbors would do something about it? |
| b. If a group of kids was spraying graffiti on a building, how likely is it that your neighbors would do something about it? |
| c. If a teenager was showing disrespect to an adult, how likely is it that your neighbors would say something? |
| d. If there was a fight in front of your home, how likely is it that your neighbors would do something about it? |
| e. If a group of kids was climbing on a parked car, how likely is it that your neighbors would say something to them? |
| f. If the local fire station was going to be closed down because of budget cuts, how likely is it that your neighbors would do something about it? |

Note: For collective efficacy measures (i.e., social cohesion, willingness to intervene), following Sampson et al. (1997), we recoded “don’t know” responses as a middle category (3) in the five-point Likert scale to signify “neither likely nor unlikely” or “neither agree nor disagree.”

**Appendix B.** Multilevel mixed-effects linear regression models predicting procedural justice and police effectiveness (matched sample)

|  | Model B1  (PJ Model) | | Model B2  (PE Model) | |
| --- | --- | --- | --- | --- |
|  | *b* | SE | *b* | SE |
| Fixed effects |  |  |  |  |
| Intercept | 2.265*** | 0.245 | 2.878*** | 0.234 |
| *Demographics* |  |  |  |  |
| **White** | **0.143***** | **0.039** | **0.054** | **0.037** |
| Female | -0.108** | 0.040 | -0.048 | 0.039 |
| Age | 0.003* | 0.002 | 0.002 | 0.001 |
| Education (Ref = Below high school) |  |  |  |  |
| Highschool diploma | 0.012 | 0.057 | 0.036 | 0.055 |
| Some college | -0.064 | 0.060 | -0.068 | 0.058 |
| Bachelor or higher | 0.055 | 0.059 | -0.063 | 0.056 |
| Homeowner | 0.091 | 0.059 | 0.077 | 0.045 |
| Work status (Ref = Full-time job) |  |  |  |  |
| Part-time job | -0.012 | 0.069 | -0.031 | 0.066 |
| Not working | 0.030 | 0.047 | 0.021 | 0.045 |
| Married | 0.012 | 0.044 | -0.021 | 0.042 |
| *Victimization/offending/self-control* |  |  |  |  |
| Victimization | 0.009 | 0.044 | -0.040 | 0.042 |
| Offending | -0.090 | 0.061 | -0.040 | 0.058 |
| Low self-control | -0.119* | 0.049 | -0.143** | 0.047 |
| *Recent experience with the police* |  |  |  |  |
| Filing complaint about the police | -0.321*** | 0.082 | -0.223** | 0.078 |
| Arrested | -0.169* | 0.073 | -0.130 | 0.070 |
| Having called the police | 0.090* | 0.043 | 0.013 | 0.041 |
| *Perceived police presence* |  |  |  |  |
| Walking officers | 0.046 | 0.026 | 0.028 | 0.024 |
| (Logged) police cars | 0.015 | 0.026 | 0.064** | 0.024 |
| *Perceived street traits/street segment type* |  |  |  |  |
| Social disorder | -0.085** | 0.031 | -0.106*** | 0.030 |
| Physical disorder | -0.076 | 0.050 | -0.070 | 0.047 |
| Social cohesion | 0.118** | 0.038 | 0.116** | 0.036 |
| Willingness to intervene | 0.057* | 0.026 | 0.036 | 0.024 |
| Fear of crime | 0.012 | 0.052 | -0.134** | 0.050 |
| Years on street segment | -0.001 | 0.002 | -0.002 | 0.002 |
| Segment type (Ref = Cold spots) |  |  |  |  |
| Cool spots | -0.059 | 0.062 | -0.046 | 0.059 |
| Drug spots | -0.008 | 0.074 | -0.021 | 0.070 |
| Violent spots | -0.070 | 0.065 | -0.068 | 0.062 |
| Drug/violent spots | -0.098 | 0.100 | -0.021 | 0.096 |
|  |  |  |  |  |
| **Random effects** |  |  |  |  |
| Individual-level variance | 0.276 | | 0.251 | |
| Street-level variance | 0.009 | | 0.008 | |

Note: n = 788 individuals (level 1); n = 342 streets (level 2); * *p* ≤ 0.05, ** *p* ≤ 0.01, *** *p* ≤ 0.001 (two-tailed); PJ = procedural justice; PE = police effectiveness; SE = standard error.

**Appendix C.** Multilevel mixed-effects ordered logit regression models predicting obligation to obey the police (matched sample)

|  | Model C1  (Model excluding PJ & PE) | | | | | Model C2  (Model Including PJ & PE) | | |
| --- | --- | --- | --- | --- | --- | --- | --- | --- |
|  | *b* | SE | OR | | *b* | | SE | OR |
| Fixed effects |  |  |  | |  | |  |  |
| Procedural justice | - | - | - | | 0.545* | | 0.213 | 1.725 |
| Police effectiveness | - | - | - | | -0.121 | | 0.217 | 0.886 |
| *Demographics* |  |  |  | |  | |  |  |
| **White** | **0.043** | **0.167** | **1.045** | | **-0.031** | | **0.167** | **0.969** |
| Female | 0.077 | 0.175 | 1.080 | | 0.132 | | 0.176 | 1.141 |
| Age | 0.008 | 0.007 | 1.008 | | 0.007 | | 0.007 | 1.007 |
| Education (ref = Below high school) |  |  |  | |  | |  |  |
| Highschool diploma | -0.099 | 0.251 | 0.906 | | -0.110 | | 0.251 | 0.896 |
| Some college | 0.077 | 0.265 | 1.080 | | 0.096 | | 0.264 | 1.101 |
| Bachelor or higher | 0.039 | 0.257 | 1.039 | | -0.017 | | 0.257 | 0.984 |
| Homeowner | 0.360 | 0.208 | 1.433 | | 0.331 | | 0.206 | 1.392 |
| Work status (ref = Full-time job) |  |  |  | |  | |  |  |
| Part-time job | -0.622* | 0.308 | 0.537 | | -0.623* | | 0.309 | 0.536 |
| Not working | -0.129 | 0.203 | 0.879 | | -0.140 | | 0.202 | 0.869 |
| Married | 0.079 | 0.191 | 1.083 | | 0.071 | | 0.191 | 1.074 |
| *Victimization/offending/self-control* |  |  |  | |  | |  |  |
| Victimization | -0.069 | 0.193 | 0.934 | | -0.076 | | 0.192 | 0.926 |
| Offending | -0.329 | 0.275 | 0.720 | | -0.292 | | 0.274 | 0.747 |
| Low self-control | -0.668** | 0.217 | 0.513 | | -0.619** | | 0.217 | 0.538 |
| *Recent experience with the police* |  |  |  | |  | |  |  |
| Filing complaint about the police | -0.341 | 0.361 | 0.711 | | -0.198 | | 0.359 | 0.820 |
| Arrested | 0.594 | 0.314 | 1.811 | | 0.657* | | 0.314 | 1.929 |
| Having called the police | 0.145 | 0.188 | 1.156 | | 0.093 | | 0.188 | 1.097 |
| *Perceived police presence* |  |  |  | |  | |  |  |
| Walking officers | 0.073 | 0.112 | 1.075 | | 0.051 | | 0.112 | 1.052 |
| (Logged) police cars | -0.101 | 0.112 | 0.904 | | -0.101 | | 0.113 | 0.904 |
| *Perceived street traits/street segment type* |  |  |  | |  | |  |  |
| Social disorder | 0.504*** | 0.137 | 1.655 | | 0.536*** | | 0.137 | 1.709 |
| Physical disorder | 0.181 | 0.216 | 1.198 | | 0.198 | | 0.213 | 1.219 |
| Social cohesion | 0.106 | 0.167 | 1.111 | | 0.069 | | 0.168 | 1.071 |
| Willingness to intervene | 0.044 | 0.111 | 1.045 | | 0.015 | | 0.111 | 1.015 |
| Fear of crime | -0.469* | 0.229 | 0.636 | | -0.485* | | 0.231 | 0.616 |
| Years on street segment | 0.001 | 0.007 | 1.001 | | 0.002 | | 0.007 | 1.002 |
| Segment type (ref = Cold spots) |  |  |  | |  | |  |  |
| Cool spots | -0.208 | 0.261 | 0.812 | | -0.187 | | 0.259 | 0.829 |
| Drug spots | -0.045 | 0.314 | 0.956 | | -0.040 | | 0.311 | 0.960 |
| Violent spots | -0.064 | 0.274 | 0.938 | | -0.039 | | 0.272 | 0.962 |
| Drug/violent spots | -0.856* | 0.434 | 0.425 | | -0.800 | | 0.431 | 0.449 |
|  |  |  |  | |  | |  |  |
| Random effects |  | | | | | | | |
| Street-level variance | 0.032 | | | 0.000 | | | | |

Note: n = 788 individuals (level 1); n = 342 streets (level 2); * *p* ≤ 0.05, ** *p* ≤ 0.01, *** *p* ≤ 0.001 (two-tailed); PJ = procedural justice; PE = police effectiveness; SE = standard error; OR = odds ratio; Threshold values indicating cut points in obligation to obey the police are not shown in the table.

**Appendix D.** KHB mediation analysis examining the indirect effect of being White individuals on obligation to obey the police via perceived procedural justice

|  | *b* | SE | OR | *p*-value |
| --- | --- | --- | --- | --- |
| Total effect | 0.025 | 0.175 | 1.026 | 0.885 |
| Direct effect | -0.031 | 0.179 | 0.969 | 0.861 |
| **Indirect effect** | **0.057** | **0.033** | **1.058** | **0.088** |

*Note*: OR = odds ratio; SE = bootstrapped standard error (5,000 iterations); *p*-values are from two-tailed test.

**Appendix E.** Rosenbaum bounds for perceived procedural justice

|  | *Γ* (Gamma) | *p*-critical |
| --- | --- | --- |
| Procedural justice model | 1.27 | 0.046 |
|  | 1.28 | 0.052 |
